# Tactical Analysis According to Age-level Groups during a 4 vs. 4 Plus Goalkeepers Small-sided Game

**DOI:** 10.3390/ijerph17051667

**Published:** 2020-03-04

**Authors:** Filipe M. Clemente, Daniel Castillo, Asier Los Arcos

**Affiliations:** 1 Escola Superior Desporto e Lazer, Instituto Politécnico de Viana do Castelo, Rua Escola Industrial e Comercial de Nun’Álvares, 4900-347 Viana do Castelo, Portugal; filipeclemente@esdl.ipvc.pt; 2 Instituto de Telecomunicações, Delegação da Covilhã, 6200-001 Covilhã, Portugal; 3 Faculty of Health Sciences, Universidad Isabel I, 09003 Burgos, Spain; 4 Physical Education and Sport Department, Faculty of Education and Sport, University of Basque Country (UPV/EHU), 01007 Vitoria-Gasteiz, Spain; asier.losarcos@ehu.eus

**Keywords:** soccer, collective behaviors, categories, centroid, stretch index

## Abstract

This study aimed to compare the collective dynamics of three different age-level groups (i.e., U13, U15 and U18) during a 4 vs. 4 plus goalkeepers small-sided game (SSG). Fifty-four male outfield soccer players aged between 13 and 18 years took part in the study. Team tactical behaviors were assessed by measuring (a) the area occupied by players of each team, (b) the distance between both teams’ centroids, (c) the players’ distance to their own team and d) the stretch index during a 4 vs. 4 plus goalkeepers SSG format. The main results revealed that larger areas were occupied by the older players (*p* < 0.001; Effect size (ES) = 0.44–0.25, small). Additionally, the mean distance between teams’ centroids was greater in older groups (*p* < 0.001; ES = 0.44–0.81, large–small). Finally, the distance between players (*p* < 0.001; ES = 0.75–0.81, moderate–large) and the stretch index (*p* < 0.001; ES = 0.44–0.47, small) were also greater in older age categories. The evidence provided in the present study might help coaches identify the influence of age on collective dynamics during SSGs and help them find task conditions that could help to improve the behaviors and positioning of younger players.

## 1. Introduction

Positive interactions among teammates and negative interactions with opponents make soccer a social system [1], with tactical behaviors being crucial for making a soccer performance successful [2]. Tactical behaviors can be assessed as individual (i.e., the player), sectorial (i.e., several players) and/or collective [2], by means of electronic performance and tracking systems [3]. Thanks to these devices, it is possible to measure collective tactical variables such as the average position of the players (i.e., the centroid), the relationship between players based on distance (i.e., the dyad) and the use of the playing space [2,3]. The analysis of these tactical variables is crucial because it allows for the assessment of the overall team organization during training tasks and match play [2]. Thus, it would be interesting for coaches to investigate the certain tactical behaviors of players during training tasks in order to understand the collective dynamics of the team.

Since soccer training strategies are mainly designed to improve individual, sectorial and collective tactical performance, tactical analyses of the training tasks have become one of the main objectives for coaches in their training context. As such, small-sided games (SSGs), which are conditioned soccer games featuring a reduced number of players and two goals [4], are frequently used in youth soccer training [5,6]. Many studies have found that SSGs are effective for maintaining and improving physical fitness performance [7,8] and promote significantly higher physical enjoyment than analytical physical training strategies [9] in young soccer players. The major research in SSGs has been focused on the quantification of internal and external training loads [7,8]; however, little literature has investigated tactical analyses in young soccer players [2]. In addition, coaching staff need greater knowledge of collective tactical behavior during training tasks in order to help them select the appropriate SSG according to the objective of the session.

Several studies have found that age determines collective tactical behavior during SSGs in young soccer players [10,11]. The oldest players occupied a greater area in comparison to young players during different SSGs [10,12,13]. Similarly, the distances between teams’ centroids were greater when older soccer players played in SSGs [10,12,13]. In addition, Olthof et al. [10] found that the mean distance between players within a team was greater among the oldest of the young soccer players during SSGs. These changes between age-groups can also be influenced by some factors such as maturation [14,15]. A study that tested the effects of maturation on tactical efficacy [16] highlighted that maturation and proficiency of peripheral perception (important contributor to making decisions) may justify a better identification of the teammates in a timely position to receive the ball, assertively predict the movements of the teammates and improve searches for new information without central vision. Moreover, the effects of accumulated experience and tactical efficacy (as discriminator of expertise) may lead to changes in exploratory behaviors of players [17]. In a study conducted on youth football players it was possible to observe that youth players with better tactical skill levels and older-age groups presented greater exploratory behaviors in games [17]. Thus, there are reasons to hypothesize that collective dynamics can be affected by age, maturation and expertise level. While a few studies have measured and compared tactical behaviors during SSGs according to age-level groups, more studies are necessary to confirm, or refute, the different collective dynamics of players in the same SSG.

Despite the importance of identifying how SSGs can influence the collective dynamics of players, studies testing the influence of small formats on the dynamics within and between teams are still scarce. Knowledge about the effects of formats on different competitive levels or age-groups is still limited (considering that different levels may react differently to the same drills/challenges). For these reasons, the aim of the study was to compare the collective dynamics of three different age groups (i.e., U13, U15 and U18) during a 4 vs. 4 plus goalkeepers small-sided game (SSG).

## 2. Materials and Methods 

### 2.1. Participants

Fifty-four male outfield soccer players aged between 13 and 18 years (age: 16.1 ± 1.9 years, height: 166.0 ± 1.0 cm, body mass: 58.77 ± 10.33 kg, body fat: 20.67% ± 7.08%, and body mass index (BMI): 21.13 ± 2.31 kg/m^2^) took part in the study (statistical power > 0.80). Participants belonged to the same soccer academy, where their teams competed at the maximum competitive level for each age level. The training hours (h) of practice per week were 4.5 h for U13, 6.0 h for U15 and 7.5 h for U18 players. The inclusion criteria were that players had to be participants of systematic training and official competitions, who had been free of muscular or skeletal injuries for at least the last month. Although goalkeepers were used as part of the study, they were excluded from the statistical analysis. The final sample consisted of 48 players, with 16 players in each age-level group (Table 1). All the participants were informed of the objectives of the research, participated voluntarily and had the possibility of withdrawing at any time without penalty. All the participants and/or their parents or tutors signed a written informed assent (players) or consent (parents). The study was conducted according to the Declaration of Helsinki, and the protocol was fully approved by the ethics committee of the university (Code: FUI1-PI002) before recruitment.

### 2.2. Procedures

The investigation was conducted over three weeks in the middle of the 2019–2020 competitive season. A previous month was used for familiarization with the study protocol, including the use of global positional system (GPS) units (WIMU PRO^TM^, RealTrack System, Almería, Spain) and the realization of the 4 vs. 4 plus goalkeepers SSG. The SSG lasted 28 min and was divided into four bouts of 4 min, with 3 min of recovery time during which players were allowed to drink fluids ad libitum. The SSG was performed on the same day of the week (i.e., Tuesday) by each age group (i.e., U13, U15 and U18), providing an interval of at least 48 h after the last high-load session (i.e., match-play) under similar weather conditions (18–22 °C, 70%–75% humidity).

Players completed the SSG on the same third-generation artificial pitch and wore their normal soccer boots. For measuring tactical variables, the players’ positions were recorded by GPS (WIMU PRO^TM^, RealTrack System, Almería, Spain). Prior to the SSG, players undertook a 20-min standardized warm-up, consisting of 7 min of slow jogging and strolling locomotion followed by 10 min of specific soccer drills and finishing with 3 min of progressive sprints and accelerations. Twenty-four hours prior to the experimental session, the players and their parents were instructed to maintain their usual habits, which included 8 h of sleep the night before each data collection session and adequate hydration and carbohydrate intake [18].

### 2.3. Data Collection and Processing

Soccer players carried GPS devices (WIMU PRO^TM^, RealTrack System, Almería, Spain) operating at a sampling frequency of 10 Hz. The technology used to collect the GPS data had been previously validated and was shown to be reliable for monitoring soccer players [19]. Participants wore a fitted body vest, and the GPS device was inserted in a purpose-built harness prior to games. To download the tactical variables, the data were transformed into raw position data (*x* and *y* coordinates). Prior to being placed on the players, the GPS devices were calibrated and synchronized following the manufacturer’s recommendations [19]. The procedure was as follows: (a) turn on the devices, (b) wait approximately 30 s after turning them on, (c) press the button to start recording once the device’s operating system is initialized and (d) analyze the data obtained from the devices using SPRO^TM^ software (RealTrack Systems, Almería, Spain). The GPS was used under good satellite conditions (number of connected satellites = 9.5 ± 0.3).

### 2.4. Tactical Variables

Team tactical behaviors were assessed by measuring (a) the area occupied by the players of each team, (b) the distance between both teams’ centroids, (c) the players’ distances to their own teams and (d) the stretch index. The effective playing area was represented by the convex hull occupied by the positions of the players on each team. This area was calculated (in m^2^) by computing the area occupied by all outfield players during the SSG, excluding the goalkeeper. Additionally, the centroid of each team was calculated using the mean values of all four outfield players’ positions (x, y) from each team in each individual frame [20]. The mean distance between each team’s centroids and the players’ distances to their own teams were then calculated over the time series [2]. Finally, the stretch index was calculated using the mean distance from each player’s position to the geometrical center (i.e., the centroid) of the corresponding team [21]. Thus, the stretch index represents the mean deviation of each player from his team’s spatial center [13]. No distinction was made between offensive and defensive phases.

### 2.5. Small-Sided Game (SSG)

Previous research used the 4 vs. 4 plus goalkeepers SSG format as a talent identification tool to determine young soccer players’ skill proficiencies [22] and as a setting for assessing the physical and physiological demands encountered by soccer players [18]. On the other hand, this SSG format was used to analyze the tactical differences among U13, U15 and U18 groups. Players completed four bouts of 4 min, each with a 3-min passive rest break between bouts aimed at minimizing the influence of fatigue [23]. The SSG pitch was 30 m long by 20 m wide (playing area = 600 m^2^ and individual interaction space = 75 m^2^ per player). The relative pitch proportions were kept constant and had the same length-to-width ratio as an official soccer field (i.e., 1.46) [24].

The teams were organized according to playing positions (i.e., one goalkeeper, one central defender, two wingers, one attacker), technical–tactical level, competitive experience and qualitative evaluation by coaches [18]. Coaches did not provide any strategic or tactical feedback during the process. All players received standardized instructions on the purpose of the game, which was to win each bout as a normal competitive match. Minor rule modifications were applied, including playing without the off-side rule, restarting the game after a goal by the goalkeeper and awarding kick-ins to the opposing side of the player who last touched the ball [18]. In addition, verbal encouragement was provided to ensure a high level of commitment from the players during the games.

### 2.6. Statistical Procedures

Results were presented as means and standard deviations (SD). The Shapiro–Wilk test (*p* > 0.05) was applied to evaluate the normal distribution of data. As such, all analyzed variables had a normal distribution and parametric analysis was used. A one-way ANOVA with a Bonferroni post hoc test was used to examine the tactical differences among age groups (i.e., U13, U15 and U18). Practical differences were calculated using Cohen’s *d* effect size (ES, trivial < 0.2; small: between 0.5 and 0.2; moderate: between 0.8 and 0.5; large: > 0.8) [25]. The data analysis was carried out using the Statistical Package for Social Sciences (SPSS 25.0, SPSS™ Inc., Chicago, IL, USA). Statistical significance was set at *p* < 0.05.

## 3. Results

**Figure 1:**
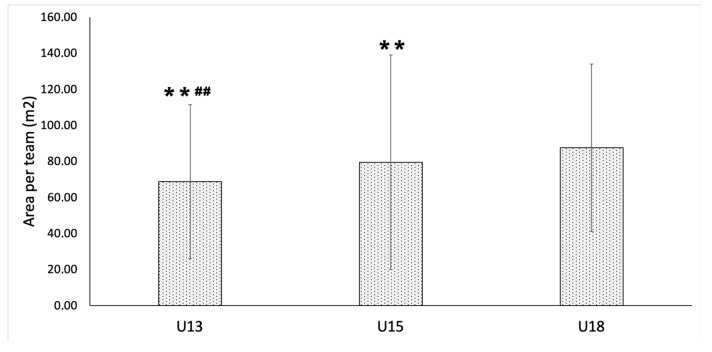
Area occupied by players of each team during the 4 vs. 4 small-sided game (SSG) plus goalkeepers according to age-level groups. ** *p* < 0.001, differences with U18 age-level group; ## *p* < 0.001, differences with U15 age-level group.

Figure 1 shows the area of each team occupied by the players during the 4 vs. 4 plus goalkeepers SSG according to age group. The U18 players used a higher area per team than the U15 (*p* < 0.001; ES = 0.44, small) and U13 (*p* < 0.001; ES = 0.25, small) players. In addition, the area occupied by U15 players during the SSG was greater than that occupied by U13 players (*p* < 0.001; ES = 0.13, trivial).

**Figure 2:**
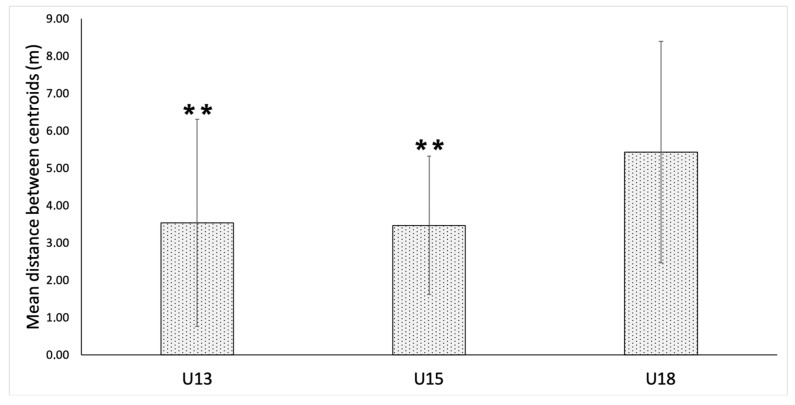
Teams’ centroid distance recorded during the 4 vs. 4 small-sided game (SSG) plus goalkeepers according to age-level groups. ** *p* < 0.001, differences with U18 age-level group.

Figure 2 presents the mean distance between the centroids of each team during the 4 vs. 4 plus goalkeepers SSG according to age group. The U18 players recorded a higher mean distance than the U15 (*p* < 0.001; ES = 0.81, large) and U13 (*p* < 0.001; ES = 0.44, small) players. However, no significant differences were reported between U15 and U13 soccer players in terms of the mean distance between centroids.

Table 2 shows the mean distance among the four players who formed each team and the stretch index during the SSG of 4 vs. 4 plus goalkeepers according to age groups. The U18 players recorded a higher mean distance in these two variables than the U15 (*p* < 0.001; ES = 0.26–0.39, small) and U13 (*p* < 0.001; ES = 0.75–0.81, moderate–large) players. In addition, the mean distance reported for these two tactical variables for U15 players was greater than that reported for U13 players (*p* < 0.001; ES = 0.44–0.47, small).

## 4. Discussion

The present study aimed to compare the collective dynamics of three different age categories during a 4 vs. 4 plus goalkeepers SSG format. This is one of the few studies to have tested the effects of age on the collective dynamics of players during SSGs, and the main results revealed that larger areas were occupied by older players. Moreover, it was also found that the mean distances between teams’ centroids were greater in older age categories. Finally, the distances between players, and the distances from players to the centroid, were also larger for players in the older age categories.

SSGs are often used by coaches to augment the perceptions of players for a given tactical issue [26], and the implications of those games on tactical behaviors and collective dynamics can be different based on the skill levels and tactical knowledge of players [10,21,27]. Likewise, assessing the technical–tactical knowledge could be an interesting strategy to understand the level of tactical awareness, decision making and skill of youth players [28]. Our study aimed to use the 4 vs. 4 plus goalkeepers format in different age groups to identify the influence of age on the area occupied by the players. The results revealed that the largest areas were occupied by U18 players (~88 m^2^), and this value progressively decreased for U15s (79 m^2^) and U13 players (69 m^2^).

This significant and progressive decrease of area occupied by the players is in line with previous studies that tested similar hypotheses for U17, U16 and U15 players in a 6 vs. 6 plus goalkeepers format [13], as well as a study that compared U13, U11 and U9 players in 3 vs. 3 plus goalkeepers and 4 vs. 4 plus goalkeepers formats [21]. Barnabe et al. [13] found that older and more experienced players exhibited greater dispersion and wider occupation while attacking, and other authors observed that declarative and procedural tactical knowledge influenced the cognitive effort of younger soccer players in making soccer performance decisions [29]. Moreover, Folgado et al. [21] found that young players tended to explore the length more than the width of the pitch, while older players tended to decrease the length per width ratio.

Occupying less area during a game can be linked to the attempt by less experienced and younger players to quickly reach the goal by swarming around the ball instead of using ball possession and positional attacks [13]. The tendency to explore width more during attacking was also revealed in a study that compared U19 and U17 players, showing that older players try to explore more opportunities from the lateral direction of the pitch [10]. As such, from a conditional perspective, younger soccer players do not have the same technical ability and experience as older ones; and consequently, their activity during the 4 vs. 4 in a 30 x 35 m playing space induces a greater physical demand due to their lack of experience [30]. The tendency to spread more or less, as reflected in the age categories of the players, should be considered by coaches when defining the length-to-width ratio of the pitch and the main objective of the game.

Our study also tested the distances between the centroids of both teams finding similar results as Aguiar et al. [31], who showed a distance of around 3 m between the team centroids in a 4 vs. 4 format played in a 40 × 30 playing space. The results revealed that the mean distance between centroids was significantly higher for U18 players (5.43 ± 2.96 m) than for U15 (3.47 ± 1.85 m) and U13 players (3.54 ± 2.77 m), although no significant differences were found between U15 and U13 players. Our results are partially in line with previous studies that compared distances between centroids for U13, U11 and U9 players [21] and between U18 and U16 players [10]. Folgado et al. [21] found that distances between centroids progressively increased as age increased from U9 to U13 for a 3 vs. 3 plus goalkeepers format and was kept relatively similar between ages for a 4 vs. 4 plus goalkeepers format. Furthermore, Olthof et al. [10] revealed that older players tend to exhibit higher correlations between centroids of both teams (more synchronized, eventually). Moreover, small differences were found in both longitudinal and lateral inter-team distances when comparing U19 and U17 age groups. These findings should also be relativized based on the pitch dimensions considering that increases in the pitch length and width can result in an increase of inter-team distances along both axes [32].

Mean distance among players per team was greater for U18 (~12.79 m) and decreased progressively to ~11.72 m (U15) and ~10.43 (U13) as age decreased. The differences between U18 and U13 players were moderate, and the remaining pairwise comparisons were small. The distances from players to the centroid were the greatest for U18 players (~9.5 m) and progressively decreased to ~8.86 m (U15) and ~7.76 m (U13) as age decreased. Moderate differences were found between U18 and U13 players, and small differences were found in the remaining comparisons. These data could be very interesting to apply on trainings; however, coaches should consider that the game status and team imbalance could influence the distance-to-team centroid with higher values when winning in superior conditions [33]. By using stretch index data to measure the spread of the players in terms of distance to the centroid, a study comparing U18 and U16 players during a 4 vs. 4 plus goalkeepers format [10] revealed greater lateral displacement in the older group. Additionally, using the stretch index, a study comparing regional- and national-level U17 players revealed that national-level players presented larger dispersion values than regional-level players [27]. Thus, it seems that older and more experienced players move farther from the team’s centroid, possibly in an attempt to explore opportunities to create imbalances among the opponents and generate space to act.

This study had some limitations. As we did not analyze moments both with and without possession of the ball, we cannot identify specific collective dynamics or measure the distances between teammates at those moments. In the future, such a methodological approach should be taken in order to improve the capacity to explain the results. Moreover, the use of just one format limits the understanding of how players behave during different SSGs. Despite that, our study is useful in terms of considering that age and possible experience might lead to different collective dynamics during SSGs, which should be considered by coaches when adjusting the task conditions to the players’ needs. A third limitation of this study is related to the non-presentation of physical demands. Such information would be useful for a mixed approach related to collective dynamics and its influence on physical demands. Such a fact should be considered in future studies.

As for practical implications, we highlight that age plays an important role in the behaviors and collective dynamics that emerged during SSGs and that, possibly, if the coach wants to increase exploration of the pitch in younger players it will be necessary to use additional conditions that may augment the perception of the players to do that, namely, using specific corridors on the width or to use small penetration zones in those zones. Future studies could consider using the same SSGs format throughout a full season to test possible variations in collective dynamics. It would also be interesting to organize the comparison by considering the declarative and processual tactical knowledge of the players.

## 5. Conclusions

This study compared the variations in the collective dynamics of players of different ages using the same SSG format. The main results of the present study revealed that older players occupy larger areas and present greater mean distances between a team’s centroids, and larger distances between players and from players to the centroid. The evidence in the present study can help coaches to identify the influence of age on collective dynamics during SSGs and possibly help them find task conditions that improve the behaviors and positioning of younger players. 

## Figures and Tables

**Table 1:** Characteristics of the soccer players according to their age-level group.

Age-level Group	Age (years)	Height (cm)	Body mass (kg)	BMI (kg/m^2^)	Body fat (%)
U13 (n = 16)	13.9 ± 0.3	157.2 ± 1.0	50.41 ± 9.57	20.50 ± 3.07	24.41 ± 5.50
U15 (n = 16)	15.7 ± 0.5	171.1 ± 1.0	60.53 ± 7.89	20.59 ± 1.85	15.94 ± 2.93
U18 (n = 16)	18.4 ± 0.8	177.3 ± 1.0	67.78 ± 6.97	21.68 ± 1.84	15.21 ± 3.27

BMI: body mass index.

**Table 2:** Mean distance recorded among the four players of each team and from players to the centroid during the 4 vs. 4 small-sided game (SSG) plus goalkeepers according to age-level groups.

Tactical Variables	U13	U15	U18	U13 vs. U15 (*p*; ES)	U13 vs. U18 (*p*; ES)	U15 vs. U18 (*p*; ES)
Mean distance among players per team (m)	10.43 ± 2.91	11.72 ± 2.76	12.79 ± 4.33	0.000; 0.44	0.000; 0.81	0.000; 0.39
Stretch index (m)	7.76 ± 2.34	8.86 ± 2.50	9.50 ± 3.87	0.000; 0.47	0.000; 0.75	0.000; 0.26

*p*: level of significance; ES: effect size (standardized effect size of Cohen).
